# Toward Building Sustainable Mars Infrastructure: A CO_2_‐Breathing Plasma Thruster for Orbit Maintenance and In Situ Oxygen Generation

**DOI:** 10.1002/gch2.202500325

**Published:** 2026-01-08

**Authors:** Anmol Taploo, Guru Sankar Duppada, Michael Keidar

**Affiliations:** ^1^ Micro‐Propulsion and Nanotechnology Laboratory, Department of Mechanical and Aerospace Engineering George Washington University Washington DC USA

**Keywords:** CO_2_‐breathing, habitat, Martian architecture, plasma, propulsion

## Abstract

This study explores a dual‐use CO_2_‐breathing plasma thruster capable of operating in very low Martian orbits (80–160 km), delivering electric propulsion and in situ oxygen generation. Experimental results demonstrate a thrust of over 1 N at input powers ranging from 0.1 to 1 kW across varying discharge frequencies. Optical emission spectroscopy reveals strong emission at 777.1 nm corresponding to atomic oxygen, along with spectral features of CO and CO_2_, confirming the effective dissociation of CO_2_ within the plasma. These findings support the viability of propulsion systems as multifunctional platforms for future Mars missions, enabling both aerial/ground mobility and human habitats without stored propellant gas.

## Graphical abstract

This study demonstrates a CO_2_‐breathing plasma thruster capable of producing high thrust and generating oxygen directly from the Martian atmosphere. By combining propulsion with in situ resource utilization, the system enables long‐duration satellite orbit maintenance and supports future human exploration. Experiments show strong thrust scaling with altitude and clear spectroscopic evidence of CO_2_ dissociation.

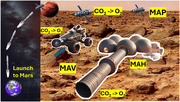

## 1 Introduction

As Earth faces escalating environmental and resource‐based pressures, the exploration of Mars has taken on renewed urgency—not merely as a scientific endeavor, but as a potential extension of human civilization. With a mean radius approximately 53% that of Earth, a surface area nearly equivalent to the total landmass of our planet, and surface gravity at 38% of Earth's, Mars offers a comparatively hospitable environment for human colonists—more so than any other nearby planetary body [1]. The vision of sending humans to Mars has evolved from a distant aspiration into a central goal of space programs globally. As humanity's ambitions extend toward a long‐term settlement on Mars, enabling technologies must evolve beyond single‐function systems to support a wide range of mission‐critical operations—propulsion, smart robotic orbiters and landers [2], life support, resource extraction, and habitat sustainability—while minimizing reliance on Earth‐based resupply. Mars’ harsh environment, with its CO_2_‐rich but low‐pressure atmosphere, extreme thermal cycles, and limited solar irradiance, necessitates the integration of dual‐function systems that can convert local resources into both mechanical utility and life‐sustaining products. To meet this challenge, there is a need to couple electric propulsion systems with in situ resource utilization (ISRU) and oxygen generation mechanisms. Studies such as those by Maiwald et al. [3], have evaluated Starship‐based Mars architectures, identified significant technological gaps, and recommended international participation in technology development. Propulsion systems have evolved from monomodal chemical designs to multimodal micro‐propulsion architectures, as demonstrated by Zolotuchin et al. [4], and iodine‐based systems that improve storability and energy density [5]. Swarms of micro‐satellites, as proposed by Levchenko et al. [2], offer scalable solutions for both orbit and surface operations, where plasma‐based propulsion systems driven by solar or compact nuclear sources could play a critical role in distributing payloads. Recent advances in Mars exploration [6] have emphasized the need for efficient ISRU technologies to support sustained robotic and human missions. A critical objective among these is the extraction of O_2_ from the CO_2_‐rich Martian atmosphere (95.9%) [7, 8, 9], not only for life support but also for potential use in propulsion. Traditional methods, such as MOXIE (Mars Oxygen In Situ Research Utilization Experiment) [10, 11], have demonstrated the feasibility of oxygen generation via solid oxide electrolysis, converting Martian atmospheric CO_2_ into O_2_ and CO at high temperatures (∼800°C). While effective, these systems typically emphasize stationary surface operations and do not address propulsion needs. Simultaneously, the scarcity of oxygen is a challenge for conventional chemical propulsion, prompting the exploration of electric propulsion (EP) systems as alternatives that can harness CO_2_ directly from the atmosphere. Although Mars presents a unique challenge for EP systems due to its low ambient surface pressure (∼6 Torr) and limited solar flux (∼40% of Earth's) [12, 13], which restricts available power. Nevertheless, utilizing CO_2_ as a propellant in a plasma‐based system provides dual benefits: thrust generation and molecular dissociation into oxygen.

Extensive experimental work has been conducted on CO_2_ plasma‐based EP and methods for oxygen generation. For plasma propulsion, Kang et al. [14] utilized a rotating gliding arc plasma source to generate CO_2_ plasma, which was then accelerated to supersonic speeds. However, they concluded that the lack of data on pressurized CO_2_ plasma posed significant challenges in achieving reliable thruster performance. Scurtu et al. [15] employed a pulsed coaxial plasma gun operating at voltages up to 2 kV, achieving pulsed thrust in the range of 11–60 N with a maximum efficiency of 35.81%. They suggested that improvement of specific impulse could be achieved using a divergent magnetic nozzle. A Hall effect thruster (HET) was studied using a CO_2_/Xe blend [9] to optimize propulsion efficiency by analyzing thrust, specific impulse, and beam characteristics. However, the system exhibited low ionization efficiency at higher power levels. Similarly, BUSEK's atmospheric‐breathing HET for Mars exploration [12] achieved an overall efficiency of 22–25%, with thrust‐to‐power (*T/P*) ratios peaking at 30 mN/kW. Zhou et al. [16] operated a surface dielectric barrier discharge (SDBD) ion wind thruster using low‐pressure CO_2_. The system demonstrated a maximum thrust of 97.48 mN/m, a *T/P* of 17.03 mN/kW, and a surface flow velocity of 23.35 m/s. However, performance was limited by partial dielectric breakdown, resulting in reduced electrical capacity due to heat buildup and space charge accumulation, which negatively affected thrust efficiency. Some propulsion system designs require a compressor or collimator in front of the thruster to compress incoming gas; however, this addition introduces drag. To address this, we previously proposed a collimator‐less concept [17, 18, 19].

In terms of O_2_ conversion from CO_2_, Hoffman et al. [10, 11] utilized solid oxide electrolysis to produce 6 g of O_2_/hr. The device was operated seven times, totaling 50 g of pure O_2_ (>98%) while consuming approximately 300 W of power. A combination of nonthermal plasmas and conducting membranes for ISRU was proposed by Guerra et al. [20], where O_2_ generation can occur without compressing CO_2_ at lower operating temperatures compared to MOXIE. A packed‐bed configuration of a DBD reactor was investigated by Ray et al. [21] where different diluent gases were tested to study the efficiency of CO_2_ decomposition. A maximum conversion rate of 19.5% was achieved, indicating concerns about limited energy efficiency in comparison with other plasma systems. In the review article, Abbas and Wali [22] compared various plasma technologies, including nonthermal plasma (NTP), DBD, microwave plasma, and gliding arc discharge for CO_2_ conversion. NTP designs show high energy efficiency with suitability for large‐scale applications. All previous designs have employed CO_2_ separately for propulsion or O_2_ conversion, but the literature lacks dual‐purpose systems. In this work, we introduce a plasma source capable of serving both functions simultaneously: propulsion and oxygen generation from CO_2_. In addition, a record‐high thrust‐to‐power ratio has been achieved.

**FIGURE 1:**
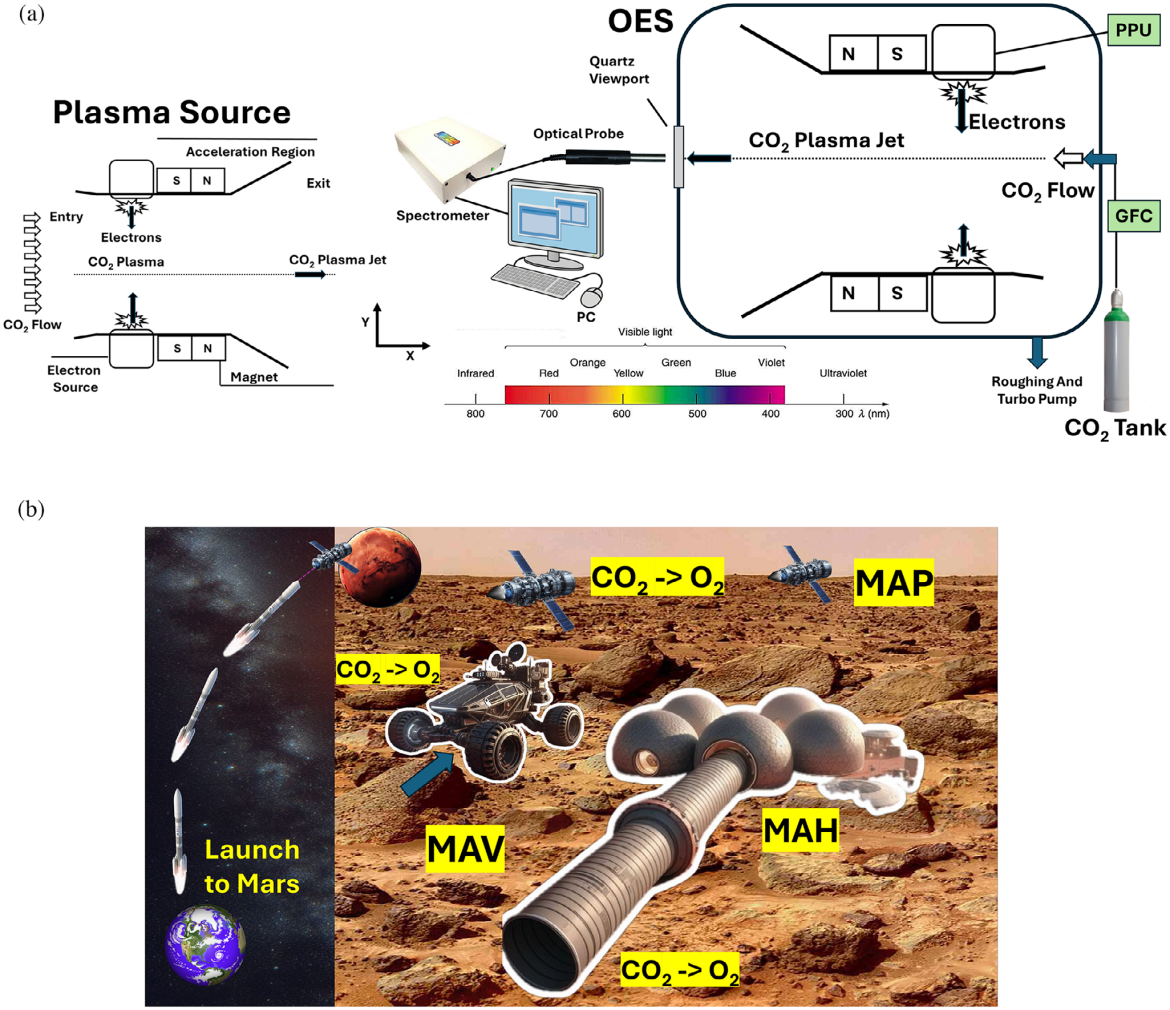
(a) The experimental setup is shown for a CBPT integrated with OES. CO_2_ is regulated via an MKS gas flow controller [25] and ionized using an electron source that is powered using a pulsed power unit (PPU). The ions generated are accelerated using the Lorentz force in the MPD acceleration stage. Emission data from the plasma is captured and analyzed to study oxygen generation via dissociation. (b) A conceptual design of CBPT‐based Martian architecture for propulsion, ground transportation, and oxygen conversion. All elements of this panel (b) were created by the authors using AI‐assisted graphical tools; no copyrighted or third‐party material was used.

Dissociation in CO_2_ discharge plasmas offers an alternative pathway to generate O_2_ while avoiding the mechanical complexity and degradation issues associated with hollow cathodes exposed to reactive species. Magnetoplasmadynamic (MPD) accelerators (thrusters) are well‐suited for such environments, offering high thrust densities [23] and compatibility with reactive gases while eliminating the need for separate neutralizers [24]. In this paper, we propose a novel MPD‐based CO_2_‐breathing plasma thruster (CBPT)/plasma source capable of dual‐function operation in very low Martian orbits (VLMO), between 80–160 km (Figure 1a). CBPT doesn't require onboard propellant storage and collimator, enabling robust and scalable operations for extended‐duration missions. Our work investigates the operational characteristics of this dual‐mode thruster, focusing on thrust generation and oxygen chemistry under variable discharge and altitude conditions. To that end, we conducted a series of experiments to characterize the thruster's performance across multiple parameters: thrust was measured as a function of applied acceleration voltage, CO_2_ mass flow rate (simulating Martian atmospheric altitudes), and source pulsing frequency. Power consumption was obtained to determine the *T/P*. To investigate the dissociation mechanisms within plasma, Optical Emission Spectroscopy (OES) was employed. The resulting spectral data provided insights into the CO_2_ breakdown pathways and oxygen species formation, validating the system's dual‐function capability.

Figure 1b provides a conceptual overview of the multi‐platform applicability of CBPT for future Mars missions, emphasizing its dual capability for propulsion and in situ oxygen generation. The visual captures a fully integrated mission architecture in which CBPT systems operate not only in space but also across the Martian surface and within human habitats. In VLMO, MAP (Mars Atmosphere Propulsion) satellites utilize atmospheric CO_2_ for sustained EP and oxygen generation, enabling long‐duration orbital science, high‐resolution imaging, and communication relay missions without carrying stored propellant. On the surface, MAV (Mars Atmosphere Vehicle) platforms—such as rovers or aerial vehicles—can employ CBPTs for mobility and localized ISRU when sufficient onboard power is available. The minimum practical power required for short missions with two crew members, up to ISRU architectures, can range [26] from 10s kW (kilowatts) to MW (megawatts). To address the kW‐MW power generation, carbon‐neutral, reliable, and long‐lasting alternatives can be explored. Imre et al. [27] suggest that nuclear power systems are well‐suited for long‐duration operation on Mars, as they remain unaffected by harsh environmental conditions such as intermittent dust storms, low surface temperatures, and the planet's thin atmosphere, while reliably generating megawatts of stable power. However, a significant challenge is the limited availability of essential reactor coolants like water and helium on Mars. Alternatively, Mane [28] evaluates solar power and regenerative fuel cell technologies. For solar power, panels must be engineered to withstand reduced sunlight intensity and include dust mitigation features. Regenerative fuel cells offer the advantage of storing energy during peak production periods and supplying it during high‐demand phases, ensuring continuous power availability. Furthermore, MAH (Mars Atmosphere Habitat) structures incorporate CBPT‐based dissociation units to continuously convert atmospheric CO_2_ into oxygen, supporting life‐support systems and reducing reliance on transported consumables. The high power generated can be transmitted via laser‐beam power [17, 29] to MAP, MAV, and MABs for scalability. Figure 1b underscores the central role of CBPT technology in supporting a closed‐loop ISRU ecosystem on Mars, where ambient CO_2_ serves for both propulsion and breathable oxygen. With the ability to scale this system across altitude regimes and operational platforms, ranging from orbital propulsion to ground transportation units and stationary habitat positions, CBPT serves as an enabler for long‐term human presence on Mars. This minimizes the need for fuel and oxygen resupply from Earth, which could take almost a year to arrive at Mars.

## 2 Plasma Diagnostics

A combination of mechanical and optical diagnostic tools was employed to characterize the performance of the CO_2_‐breathing plasma thruster. These diagnostics enable simultaneous assessment of thrust generation, electrical power consumption, and plasma chemistry related to CO_2_ dissociation.

## 3 Thrust Stand and Power Consumption

To perform thrust experiments, we have utilized our Micro‐Propulsion and Nanotechnology's (MPNL) torsional beam‐based [30, 31], which has over 90% measurement accuracy. The thrust stand was modified to measure thrust more than 1N with uncertainties within ±10%. The measurements were repeated 3 times and averaged. This type of thrust stand is comprised of a long bar oriented parallel to the ground, which makes it perpendicular to the local gravitational field. Two torsional springs (Riverhawk 5010–400) are integrated into the thrust bar to provide restoring torque, serving as the mechanical resistance to any applied forces. When a force acts on the thrust bar, it rotates until the restoring torque from the spring balances the applied force. One end of the thrust bar features a calibrator used to apply known forces, enabling the collection of deflection measurements and the creation of a force–deflection curve. The thruster is mounted on the opposite end of the bar. During operation, the thrust‐induced deflection is measured and compared against the calibration curve to determine the force produced by the thruster. Additional details regarding the thrust stand are mentioned in ref. [30, 31]. For the calibration curve, measurement of the electrostatic force was done with the help of a high‐precision scale. The scale used was a Sartorius CPA225D precision scale with a resolution of 0.01 mg (for masses up to 100 g). Force vs voltage plots were obtained, and a force vs displacement relation was created. For these experiments, the weight of the thruster was too large to be supported on the thrust stand. Consequently, the thrust was measured indirectly by attaching a lightweight plate to the movable torsional arm of the stand [31]. The control was measured from the neutrals, indicating the force of momentum of the neutral gas (thruster off) hitting the plate. The deflection observed using a PHILTEC DMS (Displacement Measurement System) sensor was averaged using a fifth‐order low‐pass Butterworth filter. A Python script was written for averaging the displacement waveform and the thrust results. The power measurements were conducted by obtaining discharge current and voltage waveforms using an HV probe and a Rogowski coil on the oscilloscope. The experimental electrical *P_exp_* power (electron source + MPD power) was calculated [31] using Equation (1):

(1) $$P_{exp}=f_{D}\left({ɡ}_{D}I_{D}V_{D}+{ɡ}_{MPD}I_{MPD}V_{MPD}\right)$$

where, *f_D_* (pulsing frequency Hz), Ʈ_*D*_/Ʈ_*MPD*_ (electron source/MPD discharge pulse width s), *I_D_*/*I_MPD_* (electron source/MPD discharge current A) and *V_D_*/*V_MPD_* (electron source/MPD discharge voltage V) are the parameters used for calculations. Based on the thrust results, *T/P* was computed.

## 4 OES for CO_2_ Plasma

This schematic (Figure 1a) illustrates the configuration used for performing non‐intrusive plasma diagnostics through OES. A fiber‐optic probe is positioned near the exit of the MPD cone (outside the plasma chamber, next to the quartz window) to collect the light emitted by excited atomic and molecular species in the plasma. This collected radiation is guided into a grating‐based spectrometer (pre‐calibrated), where it is dispersed and displayed as an intensity vs. wavelength spectrum on a computer using SpectraWiz software. The visible emissions are primarily caused by electronic transitions in heavy particles (neutrals and ions), excited through electron impact processes leading to avalanche ionization. These transitions correspond to characteristic wavelengths in the visible spectrum (as shown in the wavelength chart below for Figure 1a), and provide insight into plasma composition, temperature, and excitation mechanisms. 3 samples of the spectrum were collected and averaged with over 95% accuracy. The measurement resolution of the spectrometer was 0.1 nm. The spectral lines observed are shaped by various broadening effects—Doppler, pressure, Stark, and Zeeman—which inform the local plasma conditions.

## 5 Results

In this section, the operational behavior of the CO_2_‐breathing plasma thruster was evaluated through a series of thrust, *T/P*, and OES measurements conducted under VLMO conditions. These diagnostics enable a comprehensive assessment of both the momentum transfer performance and the underlying plasma chemistry responsible for CO_2_ dissociation. The following subsections present the measured thrust and thrust‐to‐power characteristics, followed by OES results that reveal the formation of oxygen‐bearing species within the discharge.

### 5.1 Thrust and *T/P* Performance

**FIGURE 2:**
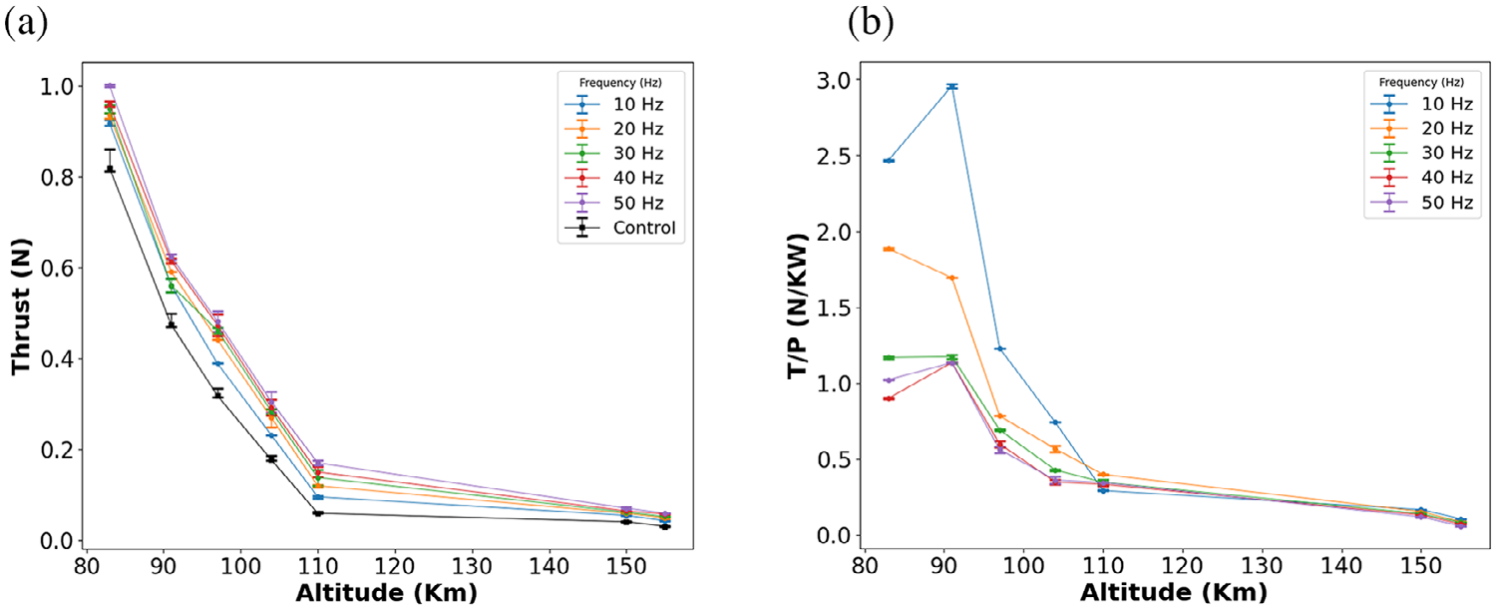
(a) The measured thrust and (b) *T/P* are shown under varying altitudes. These altitude conditions relate to specific gas flow rates within the Martian atmosphere. The data were derived by comparing the mass flow rate for a thruster channel (0.06m diameter), utilizing Mars's atmospheric model [32, 33], alongside the known input gas mass flow rate observed in the plasma chamber. This comparison is crucial, as the inlet flow velocities differ between the plasma chamber and the Martian atmosphere; nonetheless, the mass flow rate must remain conserved.

CO_2_ plasma was generated at various gas flow rate conditions by simulating Martian atmosphere altitudes. Figure 2 presents the thrust and *T/P* performance of the CBPT as a function of altitude for different pulsing frequencies for CBPT at 125 V acceleration voltage. The control flow shown in the plots indicates the force of momentum of the neutral gas (thruster off) acting on the plate. It increases with gas flow rates (decrease in altitude) from 0.03–1.05N. Figure 2 focuses on the higher altitude regime, representative of altitudes around 150–155 km and lower altitudes of 80–110 km, simulating denser atmospheric layers below 120 km. In both regimes, the thrust (Figure 2a) consistently increases with decreasing altitude and increasing pulsing frequency, indicating strong ionization. At higher altitudes, thrust output rises from ∼0.03 to 0.07 N as the frequency increases from 10 to 50 Hz. A similar positive trend is observed at lower altitudes, with thrust reaching over 1 N at 83 km and 50 Hz. This behavior confirms that pulsing enhances plasma acceleration and energy transfer, resulting in greater thrust even under rarefied gas conditions (higher altitude). The increase in thrust with frequency is attributed to higher repetition of power delivery into the plasma, allowing for more effective ionization and momentum transfer per unit time. At lower altitudes, the denser gas environment improves collisional ionization rates (shorter mean free path) and thrust output. However, it should be noted that while thrust increases, this does not necessarily imply improved efficiency, which is examined in Figure 2b. Importantly, the control exhibits the lowest thrust at all altitudes, establishing the superiority of pulsed‐mode MPD discharge for low‐density CO_2_ operation. This validates the viability of CBPTs for continuous operation in very low Mars orbits and opens doors for its application across various altitudes depending on mission requirements.

Figure 2b shows the *T/P* of CBPT under varying altitudes and pulsing frequencies, offering insight into the system's energy efficiency across operating conditions. At higher altitudes (Figure 2b), the *T/P* ratio is highest for lower pulsing frequencies, with 10 Hz consistently yielding the best efficiency, achieving up to 0.162 N/kW at 0.0065 Torr. As the frequency increases to 50 Hz, the *T/P* ratio drops, despite higher thrust being observed in Figure 2a. This trend underscores a trade‐off: while higher frequencies improve thrust magnitude, they also increase power consumption disproportionately, thereby reducing overall efficiency. At lower altitudes (Figure 2d), this trade‐off becomes even more pronounced. The *T/P* ratio increases with a decrease in altitude up to an optimal point, peaking at around 90 km, where 10 Hz operation achieves a maximum *T/P* near 3 N/kW. Beyond this point (at 83 km), the efficiency drops for all frequencies, likely due to enhanced collisional losses. The lower frequencies (10–20 Hz) again dominate in terms of efficiency across this altitude band, while higher frequencies (40–50 Hz) yield lower *T/P* ratios, especially at the upper altitude limits. The numerical values of thrust and *T/P* with their standard error (SE) are summarized in Table 1. These results indicate that efficient operation favors lower pulsing frequencies, particularly when the goal is sustained thrust with minimal power consumption, crucial for missions constrained by limited onboard power, such as those relying on solar arrays in Martian orbit or ground power systems. Together with the thrust results, this figure highlights an important design consideration: the optimal operating point must balance thrust requirements with available power. For long‐duration or resource‐limited missions, lower frequency pulsed operation at moderate altitudes (∼90–100 km) presents a sweet spot for maximizing efficiency. Larger inlet diameters capture more CO_2_, enabling sustained thruster operation at slightly higher altitudes; however, this comes at the cost of increased structural and drag considerations. The results suggest that optimal operational performance for a CBPT is achievable below 120 km.

**TABLE 1:** Experimentally measured thrust and T/*p* values varying with frequency and altitudes.

Altitude (km)	10 Hz T (N)	10 Hz T/P (N/kW)	20 Hz T (N)	20 Hz T/P (N/kW)	30 Hz T (N)	30 Hz T/P (N/kW)	40 Hz T (N)	40 Hz T/P (N/kW)	50 Hz T (N)	50 Hz T/P (N/kW)
**155**	**0.0422** ± 0.0031	**0.1056** ± 0.0074	**0.0485** ± 0.0039	**0.0862** ± 0.0078	**0.0515** ± 0.0041	**0.0812** ± 0.0080	**0.0570** ± 0.0041	**0.0709** ± 0.0075	**0.0588** ± 0.0044	**0.0591** ± 0.0049
**150**	**0.0540** ± 0.0040	**0.1667** ± 0.0095	**0.0583** ± 0.0047	**0.1537** ± 0.0083	**0.0608** ± 0.0050	**0.1395** ± 0.0086	**0.0637** ± 0.0053	**0.1314** ± 0.0088	**0.0698** ± 0.0059	**0.1186** ± 0.0077
**110**	**0.0949** ± 0.0085	**0.2923** ± 0.0084	**0.1192** ± 0.0107	**0.3986** ± 0.0225	**0.1375** ± 0.0130	**0.3469** ± 0.0240	**0.1496** ± 0.0135	**0.3334** ± 0.0291	**0.1699** ± 0.0156	**0.3447** ± 0.0263
**104**	**0.2306** ± 0.0151	**0.7425** ± 0.0596	**0.2691** ± 0.0215	**0.5673** ± 0.0454	**0.2833** ± 0.0189	**0.4280** ± 0.0311	**0.2914** ± 0.0211	**0.3491** ± 0.0314	**0.3042** ± 0.0205	**0.3619** ± 0.0330
**97**	**0.3886** ± 0.0258	**1.2293** ± 0.0754	**0.4412** ± 0.0337	**0.7836** ± 0.0706	**0.4615** ± 0.0385	**0.6919** ± 0.0494	**0.4736** ± 0.0426	**0.5988** ± 0.0539	**0.4817** ± 0.0437	**0.5624** ± 0.0508
**91**	**0.5607** ± 0.0449	**2.9577** ± 0.2112	**0.5911** ± 0.0473	**1.6942** ± 0.1523	**0.5607** ± 0.0507	**1.1745** ± 0.0984	**0.6154** ± 0.0538	**1.1346** ± 0.1021	**0.6235** ± 0.0565	**1.1338** ± 0.0951
**83**	**0.9187** ± 0.0601	**2.4666** ± 0.1159	**0.9354** ± 0.0706	**1.8862** ± 0.1116	**0.9500** ± 0.0720	**1.1689** ± 0.0901	**0.9604** ± 0.0724	**0.8994** ± 0.0809	**1.0013** ± 0.0745	**1.0218** ± 0.0919

### 5.2 CO_2_ Plasma OES

**FIGURE 3:**
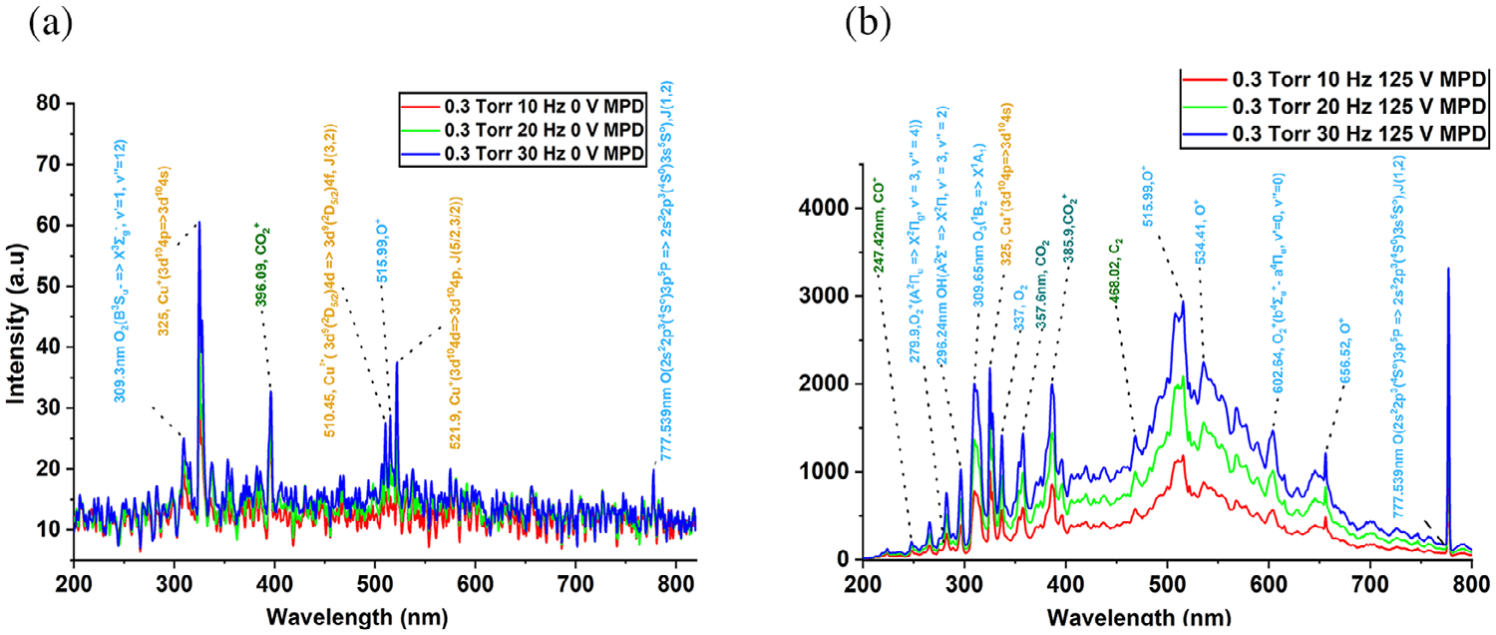
(a) OES results at 0.3 Torr, 10–30 Hz (red‐green‐blue line) pulsing frequency, and 0 V acceleration MPD voltage. (b) OES results correspond to 0.3 Torr, 10–30 Hz pulsing frequency at 125 V acceleration voltage.

The OES technique provides insight into CO_2_ plasma chemistry by studying the emissions in the visible spectrum. The emission lines are interpreted using the NIST database [34] for atoms and the molecular spectra reference [35] for molecules. Figure 3 presents the OES of the CO_2_ plasma under varying pulsing frequencies and applied acceleration voltage, highlighting the impact of discharge conditions on species formation and dissociation pathways. Figure 3a shows emission spectra at 0 V acceleration voltage for 10, 20, and 30 Hz pulse frequencies at 0.3 Torr.

The spectra display weak emission signatures, with relatively low‐intensity bands corresponding to Cu and O species (e.g., 510.6 and 777.539 nm), indicating minimal ionization or dissociation activity in the absence of applied acceleration voltage. The Cu peaks in the data arise due to emissions from the electron source of the CBPT. In contrast, Figure 3b depicts OES spectra under 125 V acceleration voltage, demonstrating a significant enhancement in emission intensity with increasing frequency, especially at 30 Hz, where plasma activity peaks. Most notably, the 777.539 nm O triplet line shows a dramatic increase in intensity, far exceeding all other spectral features. This emission originates from electronically excited atomic oxygen, formed through the dissociation of CO_2_ molecules within the high‐energy plasma environment. The prominence of the 777.1 nm peak, particularly at higher frequency and MPD settings, provides direct spectral evidence of efficient CO_2_‐to‐O_2_ conversion, validating the CBPT's potential for oxygen generation via plasma dissociation. Additionally, intensified bands of other oxygen species (e.g. 279.9 nm O_2_⁺, 309.65 nm O_3_, 337 nm O_2_, 515.99 nm O^+^, 602.64 nm O_2_^+^, and 656.52 nm O^+^) reinforce the presence of dissociation and recombined products. Additional emission features include strong bands from CO_2_ dissociation products such as 247.42 nm CO^+^, 357.6 nm CO_2_, 385.9 nm CO_2_^+,^ and 468.02 nm C_2,_ as well as atomic oxygen (O I) at 777.538 nm, which again dominates the spectrum, particularly at higher frequencies. However, the relative enhancement per volt/frequency appears more dramatic, suggesting that CO_2_ discharge conditions may be especially sensitive to pulsing frequency modulation. In conclusion, the thruster can sustain oxygen production efficiently, supporting its use in varying orbital and surface environments.

### 5.3 Statistical Analysis

All measurements in this study were subjected to standardized preprocessing, averaging, and uncertainty quantification procedures to ensure consistency and reproducibility across thrust and OES diagnostics.

- Data Preprocessing: A python script was generated to analyze thrust stand and OES results.
- Thrust Stand: Raw displacement signals from the torsional thrust stand were digitally filtered using a fifth‐order low‐pass Butterworth filter to remove high‐frequency vibration noise. Each operating condition (altitude) consisted of multiple pulsed discharges, and thrust values were computed from the averaged displacement (3 trials) response.
- OES: Emission spectra were baseline‐corrected and normalized to remove background fluctuations. For each operating condition, three independent spectra were recorded and averaged.
- Power Measurements: Voltage and current pulse waveforms were averaged over 126 values on the oscilloscope, and experiments were repeated three times at each condition.
- Data Presentation: All quantitative results (thrust, thrust‐to‐power ratio, OES intensities, and electrical measurements) are reported as: mean ± SE.
- Sample Size (n): For thrust, *T/P* and OES experiments, 3 trials were performed and data was averaged.
- Thrust Stand: Calibration was conducted by creating a Force versus Displacement plot. Thrust data achieved over 90% accuracy. The total measurement uncertainty is approximately ±10%, mainly due to displacement sensor noise, calibration mass tolerance, and variations in the mechanical damping constant.
- OES: Spectrometer had a wavelength resolution of 0.1 nm. Intensity varied across repeated spectra, reflecting good stability (<5%) of the CO_2_ discharge.
- Power Measurements: A combined uncertainty in average power <4%. The accuracy of the voltage and current probe was 1% and 2%.
- Statistical Methods: This study focuses on deterministic plasma thruster performance measurements, not comparisons between treatment groups; therefore, no t‐tests, p‐values, etc. were reported. Statistical reporting emphasizes repeatability, measurement uncertainty, and SE across repeated trials.
- Software: Thrust stand data were acquired using the DMS PHILTEC operating software, and thrust values were subsequently averaged and computed through a custom Python post‐processing script executed in Jupyter Notebook. Electrical waveforms were recorded on a digital oscilloscope and transferred to a computer for further evaluation using a Python script, where the acceleration‐stage voltage and current traces were processed to compute instantaneous power by solving Equation (1). OES measurements were obtained using SpectraWiz software, with additional preprocessing and analysis performed in Jupyter Notebook. All plots and graphical outputs were generated using OriginLab 2024.

These combined thrust, efficiency, and spectroscopic results establish the key performance characteristics of the CO_2_‐breathing plasma thruster, providing the basis for a detailed interpretation in the following discussion.″

## 6 Discussions

The dual‐mode utilization of CBPT enables both propulsion and in situ oxygen generation, presenting a unique approach to sustainable resource utilization on Mars. Across the altitude range of 80–160 km, representative of VLMO to near‐surface atmospheric conditions, the thruster demonstrated reliable performance in both thrust generation and oxygen conversion. The corresponding CO_2_ mass flow rate during these experiments was estimated between 1 × 10^−^⁵ and 1 × 10^−^⁴ kg/s (10–100 mg/s), with power consumption ranging from 0.1–1 kW. At 0.3 Torr, the power consumption by the CBPT was 300–400 W (10–30 Hz). A key indicator of the thruster's plasma chemistry is the prominent optical emission at 777.539 nm, corresponding to the O I triplet, observed at high intensity (∼3000 a.u.) in the OES spectra. Compared to other species like CO^+^ and CO_2_, the 777.5 nm line was significantly dominant, particularly under high‐frequency (30 Hz) and high‐voltage (125 V) MPD discharge conditions. This spectral behavior strongly supports the dissociation of CO_2_ into CO and O, followed by subsequent recombination into O_2_, as governed by the reactions:

(2) $$CO_{2}\to \mathrm{CO}+O$$

(3) $$O+O\to O_{2}$$

Based on these observations and conservative assessment, we estimate an oxygen conversion efficiency of 30–40%, which translates to an O_2_ mass flow rate of approximately 2.18–21.8 mg/s under typical experimental flow conditions. Even under a highly conservative scenario with only 10% dissociation, the system still delivers ∼0.7–7 mg/s of O_2_, demonstrating continuous molecular oxygen production with no additional oxidizer input. To contextualize these results, we compare them to NASA's MOXIE [10, 11], which has successfully demonstrated O_2_ generation at ∼10 g/hr (∼2.8 mg/s) at ∼300 W power (comparable with CBPT's power consumption). While MOXIE relies on solid oxide electrolysis and operates intermittently on the Martian surface with considerable mechanical complexity, CBPT achieves comparable or greater rates of oxygen production while simultaneously delivering electric propulsion. Moreover, the CBPT operates at atmospheric intake conditions, eliminating the need for gas compression hardware and enabling scalability across both orbital and surface platforms. Despite these promising outcomes, several areas for improvement and future investigation remain. First, the oxygen yield estimation based on OES intensity assumes a linear relationship between emission strength and species density, which can be influenced by electron temperature and collisional dynamics. Future work will involve quantitative calibration of OES results against mass spectrometry to refine dissociation rate estimates. Additionally, energy efficiency remains a key trade‐off: while higher frequencies enhance dissociation and thrust, they also increase power demand. Incorporating optimized pulsing schemes (e.g., duty cycle modulation) may improve both thrust‐to‐power ratios and oxygen output. Overall, these results establish the CBPT as a viable, scalable, and multifunctional ISRU solution that addresses both propulsion and life support challenges in future Mars missions. By leveraging the abundant CO_2_ in the Martian atmosphere, this system reduces payload mass, extends mission durations, and supports the foundational infrastructure for sustained human exploration.

## Author Contributions

**Anmol Taploo**: conceptualization and writing – original draft. **Anmol Taploo**, **Guru Sankar Duppada**, and **Michael Keidar**: methodology. **Anmol Taploo** and **Guru Sankar Duppada**: investigation and visualization. **Michael Keidar**: funding acquisition, project administration, and supervision. **Anmol Taploo** and **Michael Keidar**: writing – review & editing.

## Funding

Internal George Washington University, Technology Maturation Award, and National Science Foundation's National I‐Corps.

## Conflicts of Interest

The authors declare no conflict of interest.

## Data Availability

The data that support the findings of this study are available from the corresponding author upon reasonable request.

## References

[1] I. Levchenko , S. Xu , S. Mazouffre , M. Keidar , and K. Bazaka , “Space Exploration: Mars Colonization: Beyond Getting There (global challenges 1/2019),” Global Challenges 3, no. 1 (2019): 1970011, 10.1002/gch2.201970011. PMC6383964 31565356

[2] I. Levchenko , S. Xu , G. Teel , D. Mariotti , M. L. R. Walker , and M. Keidar , “Recent Progress and Perspectives of Space Electric Propulsion Systems Based on Smart Nanomaterials,” Nature Communications 9, no. 1 (2018): 879, 10.1038/s41467-017-02269-7. PMC5830404 29491411

[3] V. Maiwald , M. Bauerfeind , S. Fälker , B. Westphal , and C. Bach , “About Feasibility of SpaceX's Human Exploration Mars Mission Scenario with Starship,” Scientific Reports 14, no. 1 (2024): 11804, 10.1038/s41598-024-54012-0. 38782962 PMC11116405

[4] D. B. Zolotuchin , R. S. P. Banduru , K. P. Daniels , I. I. Beilis , and M. Keidar , “Demonstration of Electric Micropropulsion Multimodality,” Science Advances 8 (2022): 9850, 10.1126/sciadv.adc9850. PMC9451150 36070382

[5] D. Rafalskyi , J. M. Martínez , L. Habl , et al., “In‐orbit Demonstration of an Iodine Electric Propulsion System,” Nature 599, no. 7885 (2021): 411–415, 10.1038/s41586-021-04015-y. 34789903 PMC8599014

[6] C. Gross , M. Al‐Samir , J. L. Bishop , F. Poulet , F. Postberg , and D. Schubert , “Prospecting in‐situ Resources for Future Crewed Missions to Mars,” Acta Astronautica 223 (2024): 15–24, 10.1016/j.actaastro.2024.07.003.

[7] X. Chen , A. Pikalev , V. Guerra , G.‐J. Zhang , and R. Van De Sanden , “Synergy for the Plasma‐Based Co_2_ Conversion with the Solid Oxide Electrolysis Cell,” in 2024 IEEE International Conference on Plasma Science (ICOPS) (IEEE, 2024), 10.1109/ICOPS58192.2024.10627108.

[8] A. Scurtu , D. Ticoş , M. L. Mitu , C. Diplașu , N. Udrea , and C. M. Ticoș , “Splitting CO_2_ in Intense Pulsed Plasma Jets,” International Journal of Molecular Sciences 24, no. 8 (2023): 6899, 10.3390/ijms24086899. 37108062 PMC10138345

[9] Y. Yang , S. Zhou , S. Yuan , et al., “Optimization Design and Mechanism Study of Ion Thruster Performance Based on CO_2_/Xenon Multi‐Propellant,” Acta Astronautica 228 (2025): 149–157, 10.1016/j.actaastro.2024.12.008.

[10] J. A. Hoffman , M. H. Hecht , D. Rapp , et al., “Mars Oxygen ISRU Experiment (MOXIE)—Preparing for human Mars exploration,” Science Advances 8, no. 35 (2022): abp8636, 10.1126/sciadv.abp8636. PMC9432831 36044563

[11] M. Hecht , J. Hoffman , D. Rapp , et al., “Mars Oxygen ISRU Experiment (MOXIE),” Space Science Reviews 217, no. 1 (2021): 9, 10.1007/s11214-020-00782-8.

[12] V. Hruby , O. Pote , R. Szabo , and K. Hohman , Atmospheric Breathing Electric Thruster for Planetary Exploration (Busek Co. Inc, 2012).

[13] T. W. Kerslake and L. L. Kohout , “Solar Electric Power System Analyses for Mars Surface Missions,” in 34th Intersociety sponsored Energy Cleveland, Conversion Ohio Engineering by the Society of Automotive Vancouver (Glenn Research Center, 1999), https://ntrs.nasa.gov/api/citations/19990063893/downloads/19990063893.pdf.

[14] H. Kang , J. Lee , K.‐T. Kim , Y.‐H. Song , and D. H. Lee , “Conceptual Demonstration of Martian Atmosphere‐Breathing Electrical Supersonic Thruster with CO_2_‐Based Rotating Gliding Arc,” Acta Astronautica 200 (2022): 196–200, 10.1016/j.actaastro.2022.07.058.

[15] A. Scurtu , D. Ticoș , N. Udrea , M. L. Mitu , and C. M. Ticoș , “Thrust of a Pulsed Plasma Jet Measured From Deviations of a Ballistic Pendulum,” Physica Scripta 99, no. 9 (2024): 095607, 10.1088/1402-4896/ad6b4d.

[16] L. Zhou , Z. Yang , L. Wei , J. Li , H. Li , and Y. Ding , “Characteristics of a Low‐Pressure Carbon‐Dioxide Surface Dielectric Barrier Discharge Plasma Ion‐Wind Thruster,” AIAA Journal 61, no. 11 (2023): 4828–4838, 10.2514/1.J063049.

[17] L. Pekker and M. Keidar , “Analysis of Airbreathing Hall‐Effect Thrusters,” Journal of Propulsion and Power 28, no. 6 (2012): 1399–1405, 10.2514/1.B34441.

[18] A. Taploo , L. Lin , and M. Keidar , “Air Ionization in Self‐Neutralizing Air‐Breathing Plasma Thruster,” Journal of Electric Propulsion 1 (2022): 25, 10.1007/s44205-022-00022-x.

[19] A. Taploo , V. Soni , and H. Solomon , “Characterization of a Circular Arc Electron Source for a Self‐Neutralizing Air‐Breathing Plasma Thruster,” Journal of Electric Propulsion 2 (2023): 21, 10.1007/s44205-023-00058-7.

[20] V. Guerra , T. Silva , N. Pinhão , et al., “Plasmas for In Situ Resource Utilization on Mars: Fuels, Life Support, and Agriculture,” Journal of Applied Physics 132, no. 7 (2022): 070902, 10.1063/5.0098011.

[21] D. Ray , R. Saha , and S. Ch , “DBD Plasma Assisted CO_2_ Decomposition: Influence of Diluent Gases,” Catalysts 7, no. 9 (2017): 244, 10.3390/catal7090244.

[22] A. H. Abbas and W. A. Wali , “CO_2_ Conversion Based on Plasma Technologies,” Journal of Computer Science and Engineering Research 1 (2024): 7.

[23] M. R. La Pointe , “High Power MPD Thruster Performance Measurements,” in 40th Joint Propulsion Conference and Exhibit Cosponsored by AIAA, ASME, SAE, and ASEE (Glenn Research Center, 2004), https://ntrs.nasa.gov/api/citations/20040139544/downloads/20040139544.pdf.

[24] B. B. Donahue and J. B. Pearson , “Advanced Plasma Propulsion for Human Missions to Jupiter,” AIP Conference Proceedings 504 (2000): 1285–1289, https://ntrs.nasa.gov/api/citations/20000013636/downloads/20000013636.pdf.

[25] “Mass Flow Controllers,” https://www.mks.com/c/mass‐flow‐controllers?xcid=goog‐ppc‐5030&gad_source=1&gbraid=0AAAAAD_un8‐+dipE9FVYatEPE2ZDpwmjN_&gclid=Cj0KCQjwlMfABhCWARIsADGXdy9LAGCqbH8qRnqyKsMcA6yzJb9COQFAKTtdcw3JVb9jyPEiwVo_2e0aAuFUEALw_wcB.

[26] “Mars Surface Power Generation Challenges and Considerations,” https://www.nasa.gov/wp‐content/uploads/2024/01/mars‐surface‐power‐generation‐challenges‐and‐considerations.pdf?emrc=76f56b.

[27] E. A. Imre , B. Biró , and A. Aszódi , “Engineering the Energy Supply for a Martian Colony,” in 2024 9th International Youth Conference on Energy (IYCE) , (IEEE, 2024), 1–6, 10.1109/IYCE60333.2024.10634951.

[28] S. Mane , “Electricity Generation & Power Distribution on Moon & Mars,” IJMIRM 3, no. 1 (2024): 61–65.

[29] S. Sasaki , K. Tanaka , and K.‐I. Maki , “Microwave Power Transmission Technologies for Solar Power Satellites,” Proceedings of the IEEE 101, no. 6 (2013): 1438–1447, 10.1109/JPROC.2013.2246851.

[30] J. Kolbeck , T. E. Porter , and M. Keidar , “High Precision Thrust Balance Development at the George Washington University,” in 35th International Electric Propulsion Conference Georgia Institute of Technology, (George Washington, 2017).

[31] D. B. Zolotukhin , K. P. Daniels , S. R. P. Bandaru , and M. Keidar , “Magnetoplasmadynamic Two‐Stage Micro‐Cathode Arc Thruster for CubeSats,” Plasma Sources Science and Technology 28, no. 10 (2019): 105001, 10.1088/1361-6595/ab4170.

[32] “Mars Climate Database v6.1: the Web Interface,” https://www‐mars.lmd.jussieu.fr/mcd_python/.

[33] R. Modolo , S. Hess , M. Mancini , et al., “Mars‐Solar Wind Interaction: LatHyS, An Improved Parallel 3‐D Multispecies Hybrid Model,” Journal of Geophysical Research: Space Physics 121, no. 7 (2016): 6378–6399, 10.1002/2015JA022324.

[34] N.I.S.T., Ed ., “Atomic Spectra Database Lines Form,” https://physics.nist.gov/PhysRefData/ASD/lines_form.html.

[35] A. G. Gaydon and R. W. Pearse , Identification of Molecular Spectra ‐ Hardcover, (Chapman & Hall, 1976).

